# An Ex Vivo Model for the Onset of Human Labor

**DOI:** 10.1007/s43032-026-02214-4

**Published:** 2026-09-23

**Authors:** Marina Paul, Anna Paredes Barreda, Amy Gregson, Madeline King, Md Reduanul Hossain, Waleed M. Hussein, Frederick R. Walker, Roger Smith, Tamas Zakar, Jonathan W. Paul

**Affiliations:** 1 College of Health, Medicine and Wellbeing, University of Newcastle, Callaghan, NSW 2308 Australia; 2 Hunter Medical Research Institute, New Lambton Heights, NSW 2305 Australia; 3 Centre for Rehab Innovations, University of Newcastle, Callaghan, NSW 2308 Australia; 4 School of Chemistry and Molecular Biosciences, The University of Queensland, St Lucia, QLD 4072 Australia; 5 John Hunter Hospital, New Lambton Heights, NSW 2305 Australia

**Keywords:** Myometrium, Ex vivo, Models, Contraction-associated proteins, Relaxation-associated proteins

## Abstract

During gestation, the myometrium remains relaxed but undergoes a phenotypic transformation near term that initiates labor. This coincides with increased expression of contraction-associated proteins (CAPs) and decreased expression of relaxation-associated proteins (RAPs), thereby promoting the development of powerful synchronous contractions that deliver the fetus. We do not yet fully understand the biochemical signaling pathways and physiological changes that occur as human myometrium transitions from a non-laboring to a laboring phenotype. An ex vivo model for the onset of human labor can expand our knowledge. This study aimed to assess whether non-laboring pregnant human myometrium in culture undergoes a phenotypic transformation that is consistent with transitioning toward a labor-like phenotype. Term, non-laboring pregnant human myometrium biopsies were collected. A small portion was immediately snap-frozen (0 h time point), while the remaining tissue was dissected into small pieces, incubated for 48 h in serum-free medium, and then snap-frozen. Changes in the mRNA abundance of key myometrial genes were assessed by qRT-PCR, and protein levels were quantified by Western blotting. Data were analyzed using GraphPad Prism. Myometrial expression of key CAPs, including *GJA1*, *ESR1*, *PTGS2*, *NFκB1*, *NFκB3*, *IL1β*, *IL6*, and *AKR1C1,* increased significantly, while expression of the RAPs, *PLCL1*, *HSPB1*, and *HSPB6*, was decreased significantly after 48 h of ex vivo culture. Term, non-laboring pregnant human myometrial explants undergo coordinated molecular remodeling during 48 h of ex vivo culture, with many changes paralleling those reported at term labor in vivo. These findings support prolonged explant culture as a model of the molecular transition toward a labor-like state, while also demonstrating that cultured non-laboring explants may no longer faithfully represent the original non-laboring phenotype.

## Introduction

The myometrium (uterine smooth muscle) remains relaxed throughout fetal gestation, but, approaching term, undergoes a phenotypic transformation that leads to the onset of labor [1, 2]. This coincides with increased expression of contraction-associated proteins (CAPs) and decreased expression of relaxation-associated proteins (RAPs), which promote the development of powerful synchronous contractions that deliver the fetus. We do not yet fully understand the biochemical signaling pathways and physiological changes that occur as the human myometrium transitions from a non-laboring to a laboring phenotype. This is primarily due to ethical considerations surrounding human pregnancy, which, outside clinical trials, limit human studies to in vitro investigations using cell lines (two-dimensional (2D) and three-dimensional (3D)) and ex vivo biopsied tissues.

Human cell cultures are a valuable in vitro tool for studying physiological and pathological processes. In 1984, Casey et al. [3] were the first to establish methods for isolating, culturing, and verifying human myometrial smooth muscle cells (SMCs) in traditional 2D monolayer culture. They reported that the cultured cells displayed typical features of SMCs, including a spindle-shaped or elongated morphology characteristic of smooth muscle in monolayer culture [3]. Moreover, SMCs expressed smooth muscle-specific markers, retained functional properties consistent with those of myometrial smooth muscle (e.g., contractile apparatus components), and showed no major dedifferentiation or loss of smooth muscle identity for over 1 year [3]. Nevertheless, previous studies have expressed concerns about the lifespan of cultured primary cells and have shown that cell populations can divide only a finite number of times before reaching replicative senescence [4]. To overcome the limited proliferative capacity of primary cells, researchers have created immortalized cell lines by introducing telomerase expression vectors. In 1992, Perez-Reyes et al*.* [5] immortalized human SMC lines in vitro by infecting the cells with an amphotropic recombinant retroviral vector expressing the E6 and E7 open reading frames of human papillomavirus type 16 (HPV16). These immortalized SMCs exhibited significantly increased growth rates, no signs of senescence after long-term passage and continuous culture for over 1 year (> 180 population doublings), and retained some smooth muscle characteristics [5]. Nevertheless, these immortalized SMCs also exhibited changes, including reduced size and altered expression of α-smooth muscle actin (α-SMA) filaments, typical of viral oncogene-based immortalization [5]. Building on this approach, Monga et al*.* [6] subsequently established PHM1-41, a clonal immortalized cell line derived from term-pregnant human myometrium using HPV16 E6/E7. PHM1-41 cells retained morphological characteristics of proliferating SMCs and expressed smooth muscle-specific α-SMA, estrogen receptors, and oxytocin receptors (OXTR) [6]. Importantly, the cells also remained functionally responsive to oxytocin, which increased intracellular free calcium and phosphatidylinositol turnover [6]. Thus, PHM1-41 provided an immortalized model derived specifically from pregnant human myometrium while retaining several key characteristics of myometrial SMCs. To address the limitations of E6/E7 methods, such as increased genomic instability, Condon et al. [7] immortalized human non-pregnant SMCs in 2002 by retrovirally introducing the human telomerase reverse transcriptase (hTERT) gene. They found that hTERT-infected myometrial cells maintained continuous culture for over 10 months without signs of crisis or transformation, retained a spindle-shaped, elongated morphology typical of SMCs, and grew robustly under standard culture conditions. Moreover, these hTERT-immortalized SMCs exhibited a number of markers of SMCs, including α-SMA, smoothelin, *h*-caldesmon, and calponin expression, as well as genes associated with reproduction and parturition, including *OXTR*, *ESR1*, and *PGR* [7]. Soloff et al. [8] subsequently extended the hTERT approach to myometrial cells obtained from term-pregnant women, generating three telomerase-immortalized human myometrial cell lines. These cells retained a normal karyotype, expressed smooth muscle-specific α-SMA, and possessed high-affinity oxytocin-binding sites [8]. Comparison of the immortalized cells with their parental primary cultures revealed consistent changes in only approximately 1% of more than 10,000 expressed genes, while several major signaling responses, including oxytocin-stimulated ERK1/2 phosphorylation, myosin light-chain phosphorylation, and IL-1-mediated signaling, were preserved [8]. Collectively, these studies demonstrate that both oncogene- and telomerase-based immortalization can generate experimentally tractable human myometrial cell models that retain important features of the smooth muscle phenotype; however, these models of isolated cells maintained in 2D culture do not preserve the multicellular composition, extracellular matrix, and three-dimensional tissue architecture of intact human myometrium.

More recently, 3D cell culture models have advanced significantly, enabling more accurate replication of the in vivo cellular microenvironment of tissues. These approaches encompass various techniques, including; protein-based gels, such as Matrigel and collagen, which closely mimic the composition and supportive properties of the native extracellular matrix, enabling cells to self-organize, remodel the matrix, and exhibit physiological behaviors, such as contractility [9–11]; advanced bioprinting, which enable precise control over cell positioning, formation of complex shapes like rings, expression of key markers (α-SMA, caldesmon, oxytocin receptor, connexin-43, steroid receptors), and functional contractility [12–14]; polymer scaffolds, which provide structural support, tunable mechanical properties, and tissue-like architecture to promote cell adhesion, proliferation, and 3D organization [15]; and use of microfluidic technology to model the endometrial-myometrial junction and uterine wall interactions [16]. Nevertheless, these systems do come with drawbacks, such as high costs, extended preparation times, and often the requirement for specialized equipment.

Ex vivo incubation of tissue, as small explants or strips, has gained popularity as an experimental approach for investigating myometrial biology, as it better preserves the in vivo phenotype than many other models. Georgiou et al*.* [17] performed a whole-genome transcription analysis to compare gene expression profiles of freshly biopsied non-laboring myometrium (frozen at the time of cesarean section), ex vivo myometrial explants (where the non-laboring myometrium had been incubated for 30 h as explants), primary SMC lines (passage 4), and immortalized myometrial SMCs (by transfection with hTERT). The study revealed that myometrial explants most closely resembled the original fresh, non-laboring myometrial biopsies (variation of 1,444 genes compared to fresh myometrium), followed by primary myometrial SMCs (variation in 3,840 genes compared to fresh myometrium), followed by the hTERT myometrial cells (variation in 4,603 genes compared to fresh myometrium) [17]. These results indicated that the myometrial tissue explant model was superior to primary and immortalized myometrial cell cultures for studying human parturition.

While myometrial explants show promise, they do differ from biopsied tissue. The principal component analysis done by Georgiou et al*.* [17] highlighted that the gene expression profile of myometrial explants deviated significantly from that of freshly biopsied myometrial tissue, but the significance of this variation remained unknown. Consistent with this, our group has recently shown that non-laboring myometrial explants are not immune to culture-induced changes. We found a significant decrease in *OXTR* expression and significant increases in *ESR1*, *PTGS2*, *PR-A*, and *AKR1C1* (encoding 20α-HSD, an enzyme involved in functional progesterone withdrawal) over the 48 h culture period [18–20]. Importantly, we noted that each of these changes in myometrial gene expression was consistent with those observed at the onset of labor in vivo [21–37]. Recognizing this, we hypothesized that non-laboring myometrium transitions to a ‘labor-like’ state when cultured ex vivo as explants. Accordingly, this study aimed to assess whether term, non-laboring pregnant human myometrium in culture undergoes a phenotypic transformation consistent with a transition toward a labor-like phenotype.

Targets were selected to sample complementary components of myometrial activation, including excitation and contraction (*GJA1*, the principal gap-junction protein that facilitates coordinated myometrial contractions; *OXTR*, a G-protein-coupled receptor that binds oxytocin to trigger the intracellular calcium influx necessary for initiating myometrial contractions); endocrine and prostaglandin regulation (*ESR1*, *PTGS1*, *PTGS2*, *HPGD*, *AKR1C1*, *SRD5A1*, and *MED17*); inflammatory signaling (*NFKB1*, *RELA*, *TNFα*, *IL1β*, *IL6*, and *CXCL8/IL8*); calcium and cyclic-nucleotide regulation (*PLCL1*, *PLCL2*, *TRPV4*, and *PDE4*, *PDE5*, *PDE7*, and *PDE8* family members); and stress/cytoskeletal regulation (*HSPB1*, *HSPB5*, and *HSPB6*). The panel therefore included established labor-associated markers and candidates whose regulation in human labor remains incompletely defined. In recognition of its potential to model myometrial changes at the onset of labor, we have termed this our ‘ex vivo labor model’.

## Materials and Methods

### Myometrial Tissue Acquisition

The study was approved by the Hunter and New England Area Human Research Ethics Committee (2019/ETH12330). Human myometrial samples (*n* = 30) were obtained from the upper lip of an incision in the lower uterine segment during cesarean section (CS) of singleton term pregnancies (38.3–39.5 weeks of gestation). The patients’ body mass index (BMI) ranged from 20.3 to 33.6, and none were in labor. The indications for elective CS were previous CS, placenta previa, fetal distress, or breech presentation. Women were excluded if they had received steroids prenatally. Myometrial samples were kept in ice-cold serum-free Dulbecco’s Modified Eagle Medium (DMEM) with high glucose, 2 mM L-Glutamine, 1 mM Sodium Pyruvate, 40 µg/mL Gentamicin, and 10 mM HEPES and then transferred to the laboratory for explant culture.

### Myometrial Explant Culture

Approximately 100 mg of freshly biopsied myometrial tissue from each sample was immediately snap-frozen in liquid nitrogen to serve as the 0 h baseline. The remaining tissue was dissected into approximately 2 × 2 × 2 mm explants, washed in serum-free DMEM, then incubated at 37 °C for 48 h in a humidified 95% air/5% CO_2_ incubator, with the medium refreshed after 24 h. At 48 h of incubation, the explants were snap-frozen using liquid nitrogen and stored at −80 °C for later analyses.

### RNA Extraction, Reverse Transcription, and Real-Time Quantitative PCR

RNA was extracted using TRizol Reagent (Thermo Fisher Scientific, Australia) according to the manufacturer’s protocol. Tissue homogenization was performed in TRizol Reagent using a Precellys24 homogenizer (5,000 rpm for 3 × 30 s, with 20 s intervals) (Bertin Instruments, France). Following extraction, RNA samples were further purified using the TURBO DNA-*free* kit (Thermo Fisher Scientific, Australia). RNA concentration (absorbance at 260 and 280 nm) and purity were assessed using an ND-1000 spectrophotometer (Thermo Fisher Scientific, Australia). RNA integrity was verified by agarose gel electrophoresis before and after DNase treatment, with a subset of samples additionally analyzed by TapeStation, consistently yielding RIN values of ≥ 7. Each RNA sample (0.5 µg of total RNA) was spiked with 0.5 × 10^7^ copies of Alien RNA (Integrated Sciences Pty, Australia), which served as a reference, and was reverse-transcribed using the SuperScript III First-Strand Synthesis System with random hexamer primers (Thermo Fisher Scientific, Australia). The Alien RNA transcript is an in vitro*-*transcribed RNA molecule that is non-homologous to any known nucleic acids and, as such, was used as a reference gene for these studies [18–20, 34, 38, 39]. Quantitative RT-PCR was performed using QuantStudio 6 Flex Real-Time PCR (Applied Biosystems, USA). No-reverse transcription negative controls were prepared for each sample. The final volume of each PCR reaction was 20 µL, containing 10 µL of 2 × SYBR Green PCR Master Mix (Thermo Fisher Scientific, Australia), cDNA template (corresponding to 10 ng of reverse-transcribed RNA), and cDNA-specific forward and reverse primers. For the reference gene (Alien), the final volume was 20 µL containing 10 µL of 2 × SYBR Green PCR Master Mix, cDNA template (corresponding to 10 ng of reverse-transcribed RNA), and 1.0 µL of 2.5 µM of Alien Primer Mix (with proprietary sequence). No-template control samples were included on each PCR plate to detect contamination and primer dimers. All PCR reactions, reference gene, negative controls, and no-template controls were performed in duplicate. As recommended by the manufacturer (Applied Biosystems, USA), the thermal cycling conditions consisted of an initial hold at 50 °C for 2 min, followed by 95 °C for 10 min. Amplification was then performed for 40 cycles of 95 °C for 15 s and 60 °C for 1 min, with fluorescence data collected during the 60 °C step. Following amplification, melt-curve analysis was performed at 95 °C for 15 s, 60 °C for 1 min, and 95 °C for 15 s using continuous acquisition. All temperature transitions were performed at a ramp rate of 1.6 °C/s. The same thermal cycling conditions were used for all target and reference assays. Melt curves were generated for each PCR reaction to assess the homogeneity of the amplified products. PCR amplification efficiencies were determined using LinRegPCR software (version 2021.1), which estimates amplification efficiency from the slope of the linear regression fitted to the exponential phase of each amplification curve. Reactions with Ct ≥ 35 or without a single specific melt-curve peak were classified as non-quantifiable and excluded from the study. cDNA primers (Sigma-Aldrich, USA) were designed using Primer-BLAST, optimized, and validated by confirming that a single amplicon of the appropriate size was generated. The cDNA primers used in this study are listed in Table 1.

**Table 1:** Real-time PCR primer sequences

Gene Symbol (Synonyms)	Primer sequence	Amplicon size	GeneBank number
*AKR1C1* (20α-HSD)	F: AGTTCACCGCTCGCATAA	60	NM_001353.6
	R: GGCTGTAGATAGGCTTAGTGT		
*ESR1* (ERα)	F: TGAAAGGTGGGATACGAAAAGAC	66	NM_000125.3
	R: CATCTCTCTGGCGCTTGTGTT		
*GJA1* (Cx43)	F: GGAGTTCAATCACTTGGCGTG	54	NM_000165.5
	R: GGGCACCACTCTTTTGCTT		
*HPGD* (15-PGDH)	F: GCAACAACTGAGAGACACTTTTAGAAA	59	NM_000860.6
	R: AAATGTCCAGTCTTCCAAAGTGGT		
*HSPB1* (HSP27)	F: ACGCGGAAATACACGCTGC	203	NM_001540.5
	R: TACTTGGCGGCAGTCTCATC		
HSPB5 (*CRYAB*)	F: AAACATGAAGAGCGCCAGGATGA	176	NM_001885.3
	R: ATGGGAATGGTGCGCTCAG		
*HSPB6* (HSP20)	F: TTTCGGTGCTGCTAGACGTG	115	NM_144617.3
	R: AATCCGTGCTCATCCGGG		
*IL1β*	F: GCCAATCTTCATTGCTCAAGTGTC	74	NM_000576.3
	R: AAGCCATCATTTCACTGGCGA		
*IL6*	F: TGAACCTTCCAAAGATGGCTGA	73	NM_000600.5
	R: TTCACCAGGCAAGTCTCCTCATTG		
*IL8*	F: GCTCTGTGTGAAGGTGCAGTT	146	NM_000584.4
	R: ATTTCTGTGTTGGCGCAGTG		
*MED17*	F: GTGGTGACTATGCTATTTCAGTTCG	138	NM_004268.5
	R: GCCCACGAAGATGACTCCAT		
*NFκB1* (p50)	F: GCGGAGGCATGTTTGGTAGT	72	NM_003998.4
	R: GTGGGAAGCTATACCCTGGAC		
NFκB3 (*RELA*; p65)	F: ATCTGCCGAGTGAACCGAAA	95	NM_021975.4
	R: TACACCTCAATGTCCTCTTTCTGC		
*OXTR*	F: CTGGACGCCTTTCTTCTTCGT	73	NM_000916.3
	R: GAAGGCCGAGGCTTCCTT		
*PDE4A*	F: GGATCACTACCACGCTGACG	114	NM_001111307.2
	R: AGGTCCGTGAACACTGCATCTA		
*PDE4B*	F: AGCATCTCACGCTTTGGAGT	170	NM_002600.4
	R: GTCTTTAGGAGGTCTCTTTCCTGG		
*PDE4C*	F: GCCTCCAACAAGTTCAAGCG	95	NM_000923.6
	R: CGGGAGATGTACTCGGACAC		
*PDE4D*	F: AGGAGACCGAGAGAGGGAAC	94	NM_001104631.2
	R: GTCTATGAAGCCCACCTGTGA		
*PDE5A*	F: CTCTTACATACAGCGAAGTGAAC	89	NM_001083.4
	R: TCATCAGGCACTGGTCAAAATG		
*PDE7A*	F: CTGCCATTAGAAAGCAGGCAAC R: CAGATACTCATTCTGGCGACTG	87	NM_002603.4
*PDE7B*	F: CAGGCACTTTATGCTTCAGATCGC	95	NM_018945.4
	R: AGACCCTTTCACTCCACTGCT		
*PDE8A*	F: AGACCACAGAAATCCTCGACAG	115	NM_002605.3
	R: TTCTCTATCCACCCTGCGTA		
*PDE8B*	F: ACCACTCTTCCAATGCCTACC	104	NM_003719.5
	R: CTGATCGAGGCTTCCCTTTAC		
*PLCL1*	F: AGTGTCAGAAAGCAGGGATGGA	110	NM_006226.4
	R: GTTCCAAGCAAAGCTCTCAGTTG		
*PLCL2*	F: CCTCTCCTCTGTGGCCAATC	191	NM_001144382.2
	R: CCTTGAGGGACTGAATCATCATGT		
*PTGS1* (COX1)	F: CCAGGAGCTCGTAGGAGAGAAG	66	NM_000962.4
	R: GCATCAATGTCTCCATACAATTCC		
*PTGS2* (COX2)	F: ATGTTCCACCCGCAGTACAGA	101	NM_000963.4
	R: CAGCATAAAGCGTTTGCGGTA		
*SRD5A1*	F: GTGAGTTGGGCGGGACTTTA	104	NM_001047.4
	R: CTGCATGAACAGAGGCTGGT		
*TNFα*	F: CTGCACTTTGGAGTGATCGG	144	NM_000594.4
	R: AGCTTGAGGGTTTGCTACAAC		
*TRPV4*	F: TTCTTCTTCACCAACATCAA	113	NM_021625.5
	R: ATCACCAGGACAGAGTAG		

*AKR1C1* Aldo–keto reductase family 1 member C1, *ESR1* Estrogen receptor 1, *GJA1* Gap junction alpha 1, *HPGD* 15-Hydroxyprostaglandin dehydrogenase, *HSPB1* Heat shock protein family B (small) member 1, *HSPB5* Heat shock protein family B (small) member 5 (*CRYAB*), *HSPB6* Heat shock protein family B (small) member 6, *IL1β* Interleukin 1 beta, *IL6* Interleukin 6, *IL8* Interleukin 8, *MED17* Mediator of RNA polymerase II transcription subunit 17, *NFκB1* Nuclear factor kappa-B 1, *NFκB3* Nuclear factor NF-kappa-B 3 subunit (*RELA*), *OXTR* Oxytocin receptor, *PDE4A* Phosphodiesterase 4 A, *PDE4B* Phosphodiesterase 4B, *PDE4C* Phosphodiesterase 4 C, *PDE4D* Phosphodiesterase 4D, *PDE5A* Phosphodiesterase 5 A, *PDE7A* Phosphodiesterase 7 A, *PDE7B* Phosphodiesterase 7B, *PDE8A* Phosphodiesterase 8 A, *PDE8B* Phosphodiesterase 8B, *PLCL1* Phospholipase C like 1, *PLCL2* Phospholipase C like 2, *PTGS1* Prostaglandin-endoperoxide synthase 1, *PTGS2* Prostaglandin-endoperoxide synthase 2, *SRD5A1* Steroid 5-alpha-reductase, *TNFα* Tumor necrosis factor alpha, *TRPV4* Transient receptor potential cation channel subfamily V member 4

### Protein Extraction, One-dimensional (1D) SDS-PAGE, and Immunoblotting

Protein was extracted into sodium dodecyl sulfate (SDS) extraction buffer (2% SDS, 50 mM Tris pH 6.8, 5 mM EDTA) supplemented with PhosSTOP phosphatase inhibitor (Roche, Switzerland) and Complete Mini Protease Inhibitor (Roche, Switzerland). Tissue was homogenized in SDS extraction buffer using a Precellys24 homogenizer (6,500 rpm for 3 × 60 s, with 20 s intervals), after which homogenates were incubated on a rotary mixer for 1 h at 4 °C. Homogenates were then centrifuged at 15,500 g for 15 min at 4 °C (Beckman Coulter, Australia), and supernatants were collected. Protein concentration was determined using a BCA Protein Assay Kit (Thermo Fisher Scientific, Australia).

Myometrial protein extracts (up to 50 µg per lane, due to low abundance of certain gene products/proteins in pregnant human myometrium) were loaded onto 4–12% NuPAGE gels (Thermo Fisher Scientific, Australia) and separated using a Novex Mini-Cell system (Thermo Fisher Scientific, Australia) at constant voltage (200 V for 50 min; Invitrogen). S9 Fraction from Human Liver Extract (Sigma Aldrich, USA; cat# S2442; 0.5 µg/lane), SDS protein extracts from term human placenta (20 µg/lane) and human heart (20 µg/lane) were included as positive and negative controls. Following 1D SDS-PAGE, proteins were transferred to Hybond-C nitrocellulose membranes (Amersham Biosciences, UK) using the XCell II Blot Module (Invitrogen, USA). Protein transfer was verified by Ponceau S staining and imaging using an Amersham Imager 600 (GE Healthcare, USA).

The subsequent processing of the membranes was performed on an orbital rocking platform. Membranes were blocked in 5% skim milk powder in tris-buffered saline (TBS; 500 mM NaCl, 20 mM Tris) for 1 h at room temperature. The blocking solution was decanted, and primary antibodies were applied in 5 mL of 1% skim milk powder in TBS-Tween-20 (TBS-T; 500 mM NaCl, 20 mM Tris, 0.01% Tween-20) overnight at 4 °C. The antibodies are listed in Table 2. Blots were subjected to four 5-min washes with 100 mL of TBS-T followed by horseradish peroxidase (HRP)-conjugated anti-rabbit IgG (Cell Signaling Technology, USA; cat# 7074) or anti-mouse IgG (Cell Signaling Technology, USA; cat# 7076) secondary antibody as appropriate, applied at 1∶2500 dilution in 5 mL of 1% skim milk powder in TBS-T for 1 h at room temperature. Blots were then washed three times in 100 mL of TBS-T for 5 min, and immunoreactivity was detected using Immobilon Forte (high-sensitivity) Western HRP substrate (Millipore, Australia) or Immobilon Classico Western HRP substrate (Millipore, Australia). Bands were visualized using the Amersham Imager 600 (GE Healthcare, USA). Blots were first probed with the antibody of interest, then stripped and re-probed with an antibody against β-actin to serve as a loading control. Membranes were stripped by 2 × 5 min incubations in 100 mL of 0.2 M NaOH. Stripped blots were then washed three times for 3 min in 100 mL TBS-T before being re-probed according to the outlined regimen.

**Table 2:** Primary antibodies used for western blotting

Antibody	Clonality	Host species	Dilution	Company (catalog #)
anti-β-ACTB (β-Actin)	Monoclonal	Mouse	1∶4,000	Abcam (cat# ab8226)
anti-AKR1C1 (20α-HSD)	Polyclonal	Rabbit	1∶1,000	GeneTex (cat#GTX105620)
anti-GJA1 (Cx43)	Polyclonal	Rabbit	1∶1,000	Cell Signaling Technology (cat#3512S)
anti-HSPB1 (HSP27)	Monoclonal	Mouse	1:2,500	Abcam (cat#ab2790)
anti-HSPB5 (αB-Crystallin)	Monoclonal	Mouse	1∶1,000	Stressgen (cat#SPA-222)
anti-HSPB6 (HSP20)	Monoclonal	Mouse	1∶1,000	Abcam (cat#ab125125)
anti-OXTR	Monoclonal	Rabbit	1∶1,000	Abcam (cat#ab181077)
anti-PLCL1	Monoclonal	Rabbit	1:1,000	Abcam (cat#ab157200)
anti-PTGS2	Monoclonal	Rabbit	1∶1,000	ABclonal (cat#A3560)

*AKR1C1* Aldo–keto reductase family 1 member C1 (20α-HSD), *GJA1* Gap junction alpha 1 (Connexin 43), *HSPB1* Heat shock protein family B (small) Member 1 (HSP27), *HSPB5* Heat shock protein family B (small) Member 5 (αB-Crystallin), *HSPB6* Heat shock protein family B (small) Member 6 (HSP20), *OXTR* Oxytocin receptor, *PLCL1* Phospholipase C Like 1, *PTGS2* Prostaglandin-endoperoxide synthase 2

The eight proteins selected for immunoblotting were chosen to assess transcript-protein concordance across distinct functional modules and to reflect the availability of paired tissue and antibodies that produced interpretable bands at the expected molecular weight. The panel comprised GJA1, OXTR, and PTGS2 as established labor-associated targets; AKR1C1 and PLCL1 as regulators of steroid or calcium signaling; and HSPB1 (HSP27), HSPB5 (αB-crystallin), and HSPB6 (HSP20) as stress/cytoskeletal proteins.

### Data and Statistical Analysis

All mRNA abundance data were expressed relative to the Alien reference RNA. The relative mRNA abundance was calculated as 2^−ΔCt^ using the delta Ct (∆Ct) method [40], where ∆Ct was defined as the mean Ct of the target transcript minus the mean Ct of the Alien RNA reference. To estimate the magnitude of expression change during ex vivo culture, ∆∆Ct was calculated for each participant as ∆Ct_48 h_ − ∆Ct_0 h_, and the corresponding fold-change was calculated as 2^−∆∆Ct^. Fold-changes are presented as geometric means with 95% confidence intervals calculated on the log2 fold-change scale and back-transformed, with each participant’s 0 h sample serving as the calibrator for the corresponding 48 h sample.

Densitometric analysis of immunoreactive protein bands was performed using Amersham Imager 600 analysis software (GE Healthcare, USA). The protein abundance data (optical density (OD; arbitrary units)) were expressed relative to β-actin. Statistical analyses were conducted using GraphPad Prism (USA) and confirmed with STATA (USA). All mRNA and protein relative abundance values were checked with the Shapiro–Wilk distribution test, and data that were not normally distributed were logarithmically transformed to approach normal distribution. Graphical data are presented as mean ± SEM. A paired t-test was used to compare the two groups. *P*-values < 0.05 were considered statistically significant.

## Results

### Expression of Genes Encoding Key Contraction-Associated Proteins and Regulators of Myometrial Function Following Ex Vivo Culture

**Fig. 1:**
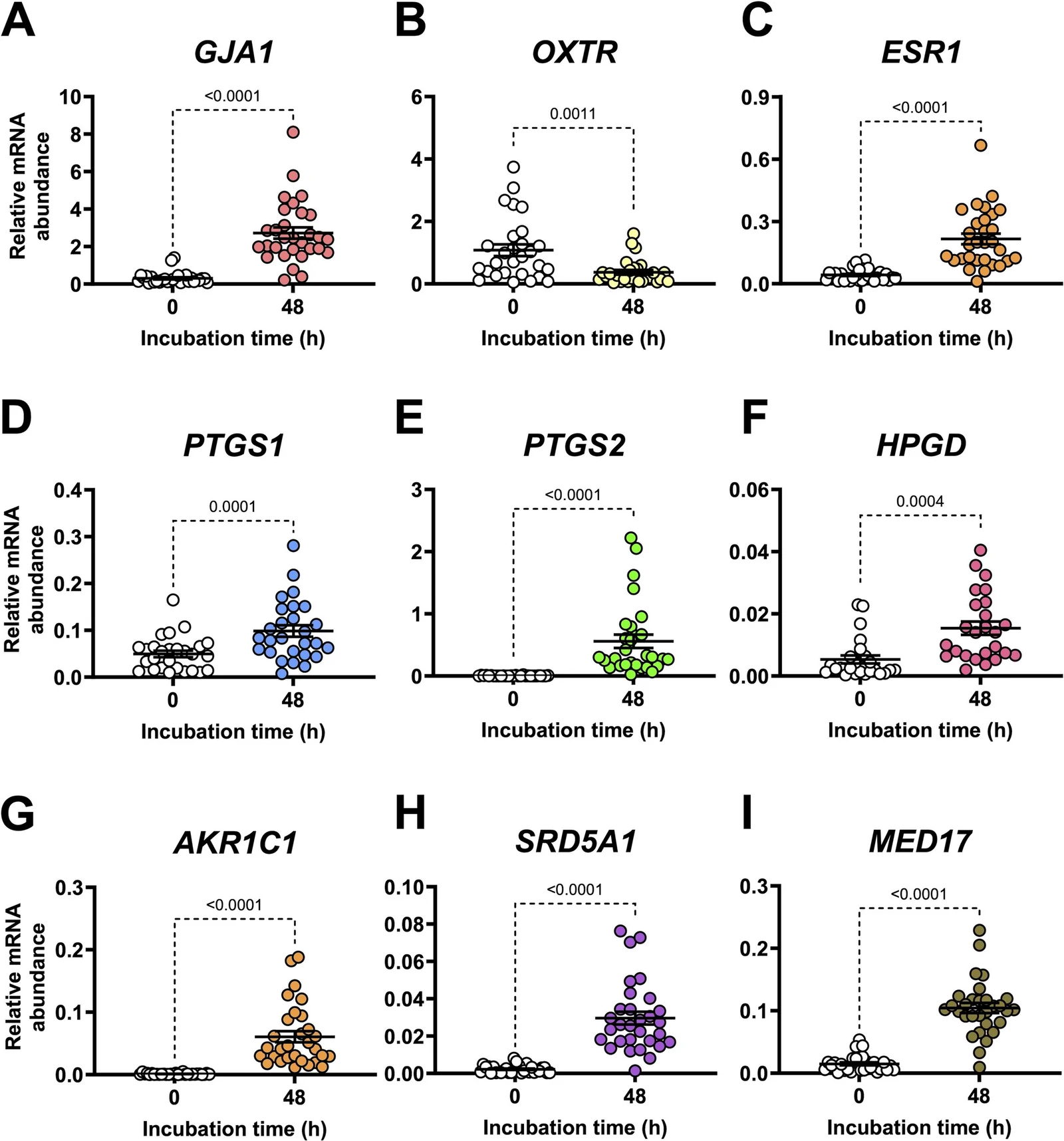
Change in the relative mRNA abundance of CAPs and their regulators when term, non-laboring pregnant human myometrium was cultured for 48 h ex vivo. Relative mRNA abundance of (**A**) *GJA1*, (**B**) *OXTR*, (**C**) *ESR1*, (**D**) *PTGS1*, (**E**) *PTGS2*, (**F**) *HPGD*, (**G**) *AKR1C1*, (**H**) *SRD5A1*, and (**I**) *MED17* was measured in myometrium frozen either immediately after biopsy (0 h) or following 48 h incubation ex vivo (*n* = 30). mRNA abundance is expressed relative to Alien reference. Data were checked for distribution using the Shapiro–Wilk normality test and analyzed by paired *t*-test. Data are mean ± SEM. A significant *p*-value is indicated

To evaluate the ex vivo labor model, we examined the expression of genes that encode key CAPs, including *GJA1*, *OXTR*, and *PTGS2* (and *PTGS1*), as well as genes encoding established regulators of myometrial function, including *ESR1*, *HPGD*, *AKR1C1*, *SRD5A1*, and *MED17*. Following the 48 h culture, there was a significant increase in the myometrial expression of *GJA1* (*p* < 0.0001), *ESR1* (*p* < 0.0001), *PTGS1* (*p* = 0.0001), *PTGS2* (*p* < 0.0001), *HPGD* (*p* = 0.0004), *AKR1C1* (*p* < 0.0001), *SRD5A1* (*p* < 0.0001), and *MED17* (*p* < 0.0001), while the expression of *OXTR* (*p* = 0.0011) was significantly decreased (Fig. 1). The magnitude of these changes varied considerably, ranging from a 1.88-fold increase in *PTGS1* to a 171-fold increase in *PTGS2*, while *OXTR* decreased to 0.35-fold of the paired 0 h baseline (Table 3).

**Table 3:** Summary of the results

Gene	48 h vs 0 h Fold Change (95% CI)	Ex Vivo Labor Model	In Vivo Labor (Non-Laboring Versus Laboring Myometrium) Changes Reported in the Literature	References
*ESR1*	5.01 (3.86–6.49)	Increased	Increased	Welsh et al. [37] Mesiano et al. [32]
*GJA1*	9.95 (6.77–14.64)	Increased	Increased	Chow and Lye [23]
*OXTR*	0.35 (0.20–0.64)	Decreased	Decreased	Bossmar et al. [21] Fuchs et al*.* [24] Rivera et al. [29] Chan et al. [36] Sharp et al. [30]
*PTGS1*	1.88 (1.35–2.62)	Increased	No change	Moore et al. [27] Slater et al. [54]
*PTGS2*	171.45 (98.69–297.85)	Increased	Increased	Chan et al. [36] Slater et al. [54]
*HPGD*	4.24 (2.18–8.26)	Increased	Unconfirmed	
*PR-A*	NA Data previously published in Ilicic et al. [19]	Increased	Increased	Mesiano et al. [32] Merlino et al*.* [26] Pieber et al*.* [28]
*PR-B*	NA Data previously published in Ilicic et al. [19]	No change	No change	Mesiano et al. [32] Merlino et al*.* [26] Pieber et al*.* [28]
*AKR1C1*	58.18 (43.27–78.24)	Increased	Increased	Paul et al. [34] Nadeem et al. [33] Nadeem et al. [35] Williams et al. [31] Lee et al. [25]
*SRD5A1*	17.30 (11.02–27.17)	Increased	Decreased	Lee et al. [25]
*MED17*	9.15 (6.21–13.50)	Increased	Unconfirmed	
*NFκB1*	12.50 (8.50–18.38)	Increased	Unconfirmed	
*NFκB3*	5.10 (3.66–7.13)	Increased	Unconfirmed	
*TNFα*	10.49 (6.72–16.40)	Increased	Increased	Chan et al. [36] Bollapragada et al*.* [74]
*IL1β*	31.00 (16.75–57.36)	Increased	Increased	Sharp et al. [30] Huang et al*.* [75] Lopez et al. [76]
*IL6*	122.70 (58.98–255.28)	Increased	Increased	Chan et al. [36] Sharp et al. [30]
*IL8*	6,138.73 (2,589.70–14,551.50)	Increased	Increased	Sharp et al. [30]
*PLCL1*	0.41 (0.28–0.61)	Decreased	Decreased	Chan et al. [36] Bollapragada et al*.* [74] Sharp et al. [30]
*PLCL2*	5.57 (3.74–8.29)	Increased	Unconfirmed	
*TRPV4*	5.21 (3.33–8.15)	Increased	Unconfirmed	
*HSPB1*	0.49 (0.34–0.70)	Decreased	Decreased (protein)	MacIntyre et al. [41]
*HSPB5*	3.74 (2.55–5.48)	Increased	Unconfirmed	
*HSPB6*	0.53 (0.36–0.77)	Decreased	Decreased	Chan et al. [36] Sharp et al. [30]
*PDE4A*	5.42 (3.73–7.86)	Increased	Unconfirmed	
*PDE4B*	27.99 (16.28–48.13)	Increased	Unconfirmed	
*PDE4C*	20.39 (11.92–34.90)	Increased	Unconfirmed	
*PDE4D*	4.57 (2.88–7.24)	Increased	Unconfirmed	
*PDE5A*	2.85 (1.80–4.51)	Increased	Unconfirmed	
*PDE7A*	9.49 (6.62–13.61)	Increased	Unconfirmed	
*PDE7B*	2.04 (1.47–2.82)	Increased	Unconfirmed	
*PDE8A*	13.23 (9.36–18.68)	Increased	Unconfirmed	
*PDE8B*	1.09 (0.75–1.60)	No change	Decreased	Chan et al. [36] Sharp et al. [30]

Fold-change values represent the geometric mean of the paired 48 h/0 h expression ratio, calculated using the 2^−ΔΔCt^ method. Values > 1 indicate increased expression and values < 1 indicate decreased expression relative to the paired 0 h baseline. Values in parentheses represent 95% confidence intervals. Sample size was *n* = 30. *PR-A* and *PR-B* results were reported previously and are included for context. ‘Unconfirmed’ indicates that no sufficiently established in vivo labor comparison could be identified; it does not indicate absence of expression or absence of change

*AKR1C1* Aldo–keto reductase family 1 member C1, *ESR1* Estrogen receptor 1, *GJA1* Gap junction alpha 1, *HPGD* 15-Hydroxyprostaglandin dehydrogenase, *HSPB1* Heat shock protein family B (small) member 1, *HSPB5* Heat shock protein family B (small) member 5 (*CRYAB*), *HSPB6* Heat shock protein family B (small) member 6, *IL1β* Interleukin 1 beta, *IL6* Interleukin 6, *IL8* Interleukin 8, *MED17* Mediator of RNA polymerase II transcription subunit 17, *NFκB1* Nuclear factor kappa-B 1, *NFκB3* Nuclear factor NF-kappa-B 3 subunit (*RELA*), *OXTR* Oxytocin receptor, *PDE4A* Phosphodiesterase 4 A, *PDE4B* Phosphodiesterase 4B, *PDE4C* Phosphodiesterase 4 C, *PDE4D* Phosphodiesterase 4D, *PDE5A* Phosphodiesterase 5 A, *PDE7A* Phosphodiesterase 7 A, *PDE7B* Phosphodiesterase 7B, *PDE8A* Phosphodiesterase 8 A, *PDE8B* Phosphodiesterase 8B, *PLCL1* Phospholipase C like 1, *PLCL2* Phospholipase C like 2, *PTGS1* Prostaglandin-endoperoxide synthase 1, *PTGS2* Prostaglandin-endoperoxide synthase 2, *SRD5A1* Steroid 5-alpha-reductase, *TNFα* Tumor necrosis factor alpha, *TRPV4* Transient receptor potential cation channel subfamily V member 4

### Expression of Inflammatory Mediators Following Ex Vivo Culture

**Fig. 2:**
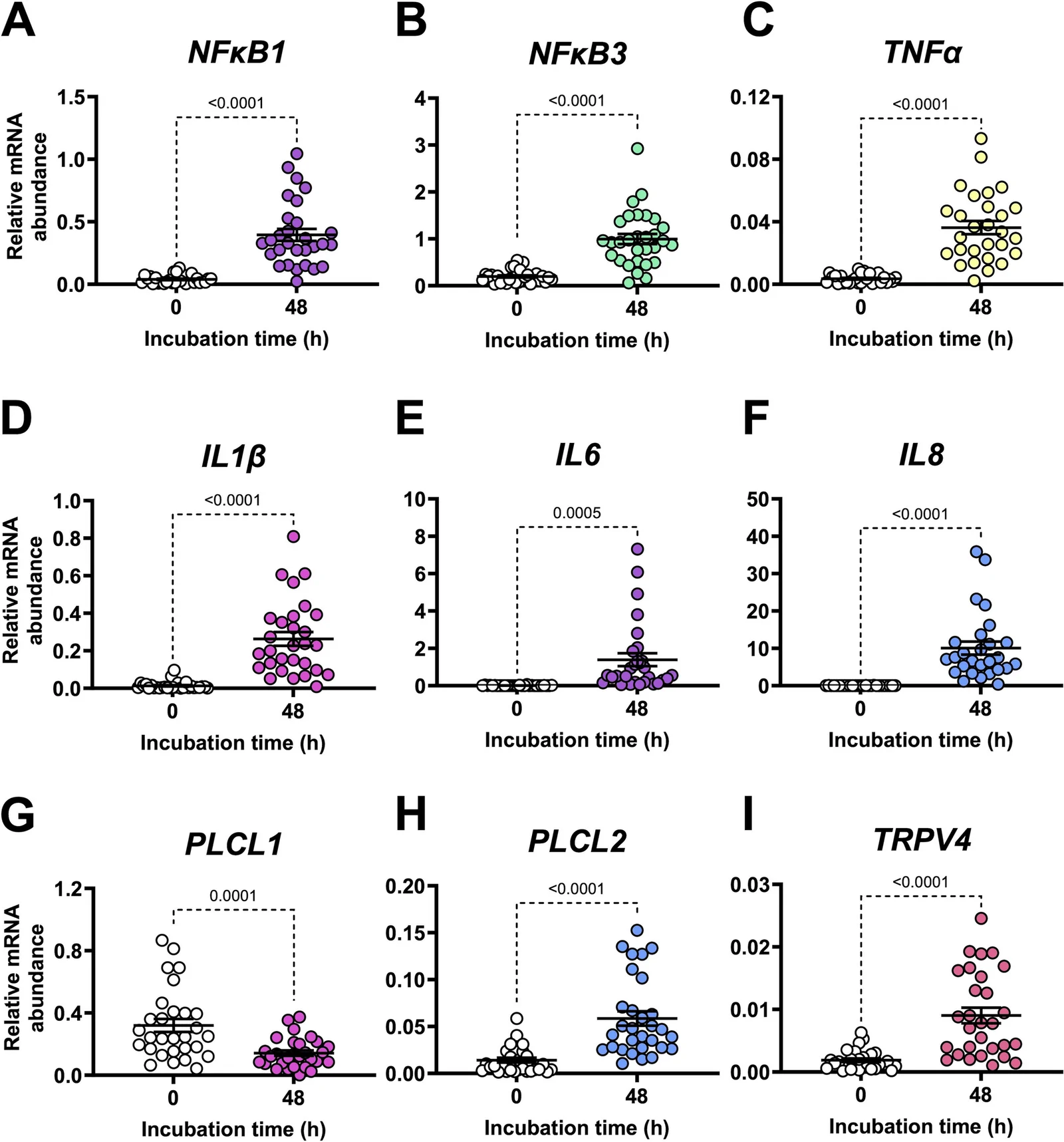
Change in the relative mRNA abundance of inflammatory mediators when term, non-laboring pregnant human myometrium was cultured for 48 h ex vivo. Relative mRNA abundance of (**A**) *NFκB1*, (**B**) *RELA*, (**C**) *TNFα*, (**D**) *IL1β*, (**E**) *IL6*, (**F**) *IL8,* (**G**) *PLCL1,* (**H**) *PLCL2,* and (**I**) *TRPV4* was measured in myometrium frozen either immediately after biopsy (0 h) or following 48 h incubation ex vivo (*n* = 30). mRNA abundance is expressed relative to Alien reference. Data were checked for distribution using the Shapiro–Wilk normality test and analyzed by paired *t*-test. Data are mean ± SEM. A significant *p*-value is indicated

As labor onset is associated with sterile inflammatory signaling, we assessed the expression of the inflammatory mediators within the ex vivo labor model. Following 48 h of culture, there was a significant increase in myometrial expression of *NFκB1* (*p* < 0.0001), *NFκB3* (*p* < 0.0001), *TNFα* (*p* < 0.0001), *IL1β* (*p* < 0.0001), *IL6* (*p* = 0.0005), *IL8* (*p* < 0.0001), *PLCL2* (*p* < 0.0001), and *TRPV4* (*p* < 0.0001), while the expression of *PLCL1* (*p* = 0.0001) was significantly decreased (Fig. 2). Culture induced particularly marked changes in several inflammatory transcripts, including 31.0-fold, 122.7-fold, and 6.14 × 10^3^-fold increases in *IL1β*, *IL6*, and *IL8*, respectively (Table 3).

### Expression of Small Heat Shock Proteins Following Ex Vivo Culture

**Fig. 3:**
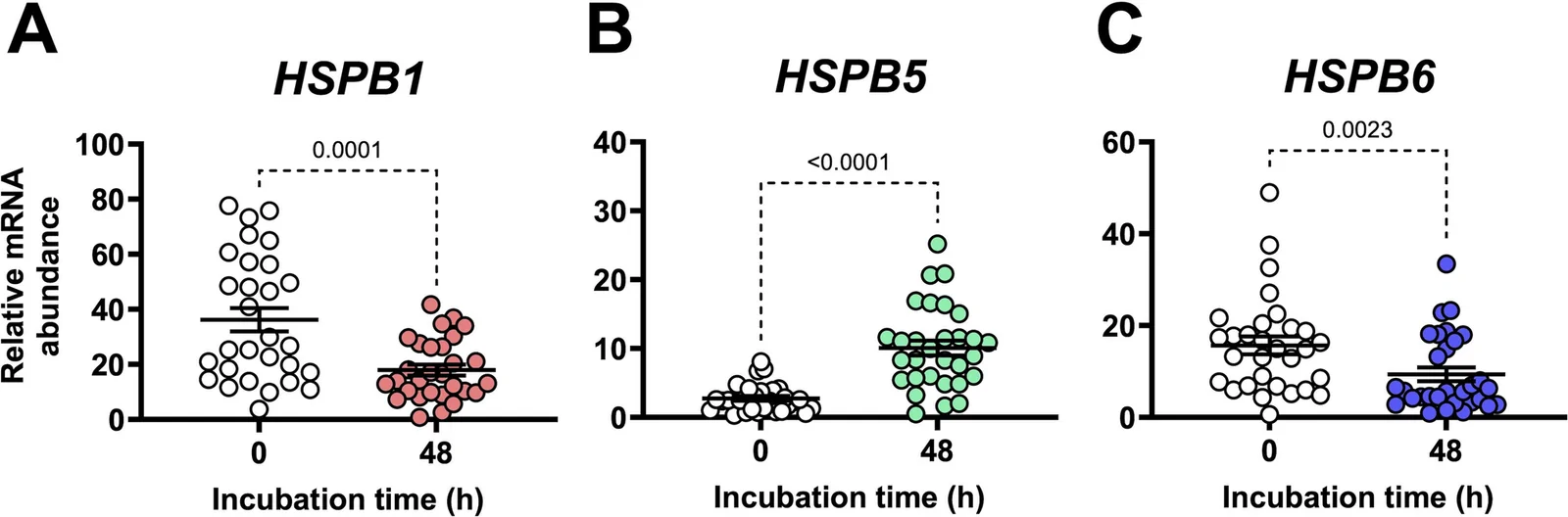
Change in the relative mRNA abundance of HSPs when term, non-laboring pregnant human myometrium was cultured for 48 h ex vivo. Relative mRNA abundance of (**A**) *HSPB1*, (**B**) *HSPB5*, and (**C**) *HSPB6* was measured in myometrium frozen either immediately after biopsy (0 h) or following 48 h incubation ex vivo (*n* = 30). mRNA abundance is expressed relative to Alien reference. Data were checked for distribution using the Shapiro–Wilk normality test and analyzed by paired *t*-test. Data are mean ± SEM. A significant *p*-value is indicated

Significant changes in small heat shock protein (HSP) levels have been documented in association with labor onset in vivo in humans [41] and rats [42, 43]. As such, the expression of HSP genes was assessed within the ex vivo labor model, including heat shock protein family B (small) member 1 (*HSPB1*), heat shock protein family B member 5 (*HSPB5*), and heat shock protein family B (small) member 6 (*HSPB6*), which encode HSP27, αB-crystallin, and HSP20, respectively. Following 48 h culture, the expression of *HSPB1* (*p* = 0.0001) and *HSPB6* (*p* = 0.0023) significantly decreased, while the expression of *HSPB5* (*p* < 0.0001) significantly increased (Fig. 3). *HSPB5* increased 3.74-fold, whereas *HSPB1* and *HSPB6* decreased to 0.49- and 0.53-fold of baseline, respectively (Table 3).

### Expression of Phosphodiesterase Isoforms During Ex Vivo Culture

**Fig. 4:**
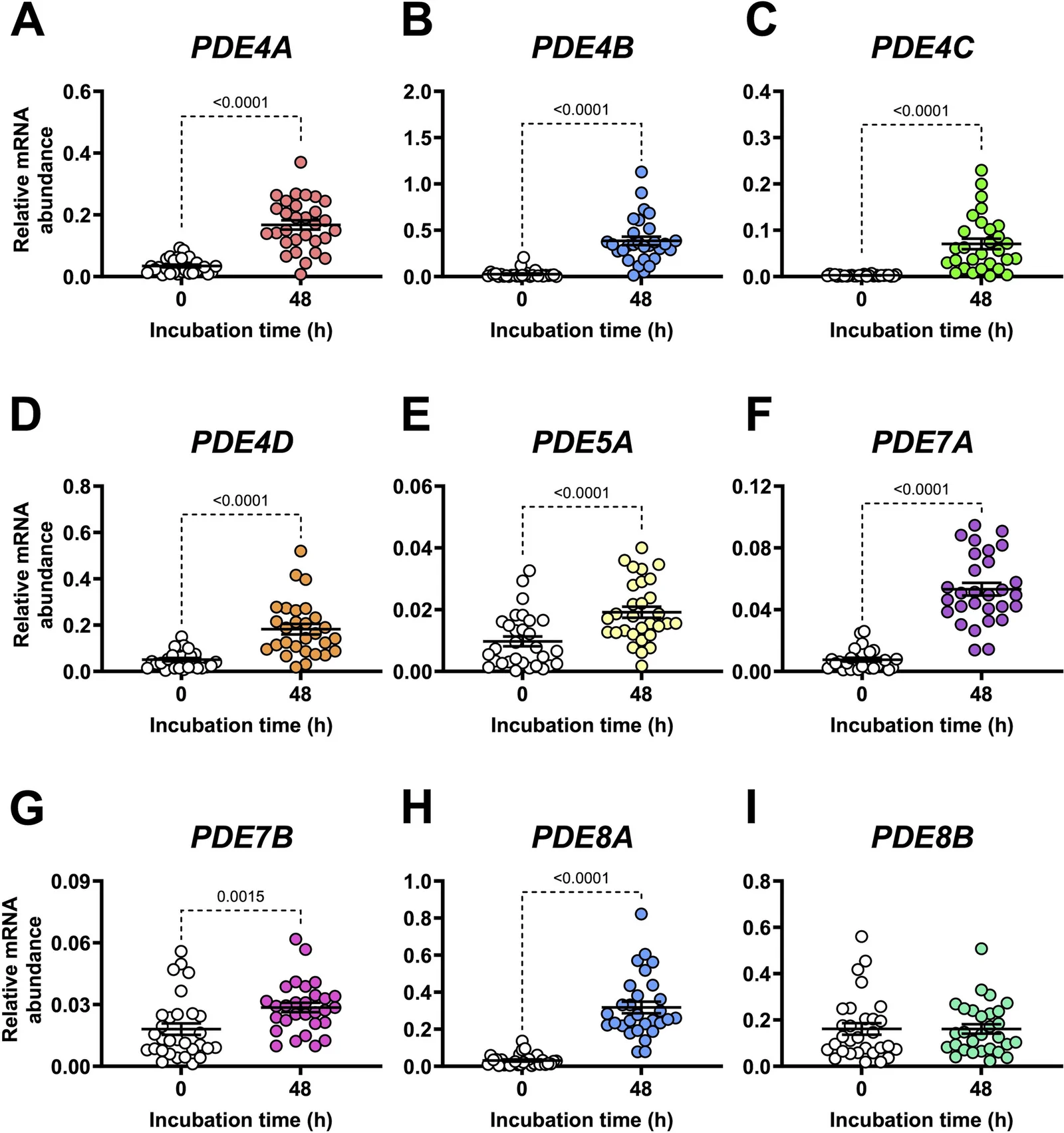
Change in the relative mRNA abundance of phosphodiesterase isoforms when term, pregnant non-laboring human myometrium was cultured for 48 h ex vivo**.** Relative mRNA abundance of (**A**) *PDE4A*, (**B**) *PDE4B*, (**C**) *PDE4C*, (**D**) *PDE4D*, (**E**) *PDE5A*, (**F**) *PDE7A,* (**G**) *PDE7B,* (**H**) *PDE8A,* and (**I**) *PDE8B* was measured in myometrium frozen either immediately after biopsy (0 h) or following 48 h incubation ex vivo (*n* = 30). mRNA abundance is expressed relative to Alien reference. Data were checked for distribution using the Shapiro–Wilk normality test and analyzed by paired *t*-test. Data are mean ± SEM. A significant *p*-value is indicated

PDE is a family of enzymes that break down cyclic nucleotides like cyclic adenosine monophosphate (cAMP) and cyclic guanosine monophosphate (cGMP). These molecules act as second messengers in cells helping regulate relaxation and contraction. Accordingly, we examined the expression of various PDE isoforms in the ex vivo labor model. Following 48 h culture, there was a significant increase in myometrial expression of *PDE4A* (*p* < 0.0001), *PDE4B* (*p* < 0.0001), *PDE4C* (*p* < 0.0001), *PDE4D* (*p* < 0.0001), *PDE5A* (*p* < 0.0001), *PDE7A* (*p* < 0.0001), *PDE7B* (*p* = 0.0015), and *PDE8A* (*p* < 0.0001), while the expression of PDE8B (*p* = 0.9029) remained unchanged (Fig. 4). Among the PDE isoforms, fold increases ranged from 2.04-fold for *PDE7B* to 28.0-fold for *PDE4B*, whereas *PDE8B* remained essentially unchanged at 1.09-fold of baseline (Table 3).

### Assessment of Protein Level Changes within the Ex Vivo Model

Changes in gene expression often correlate with changes in protein level; however, the relationship is not necessarily one-to-one. As such, many genes that revealed significant changes in mRNA abundance within the ex vivo model were also assessed at the protein level by Western blotting.

**Fig. 5:**
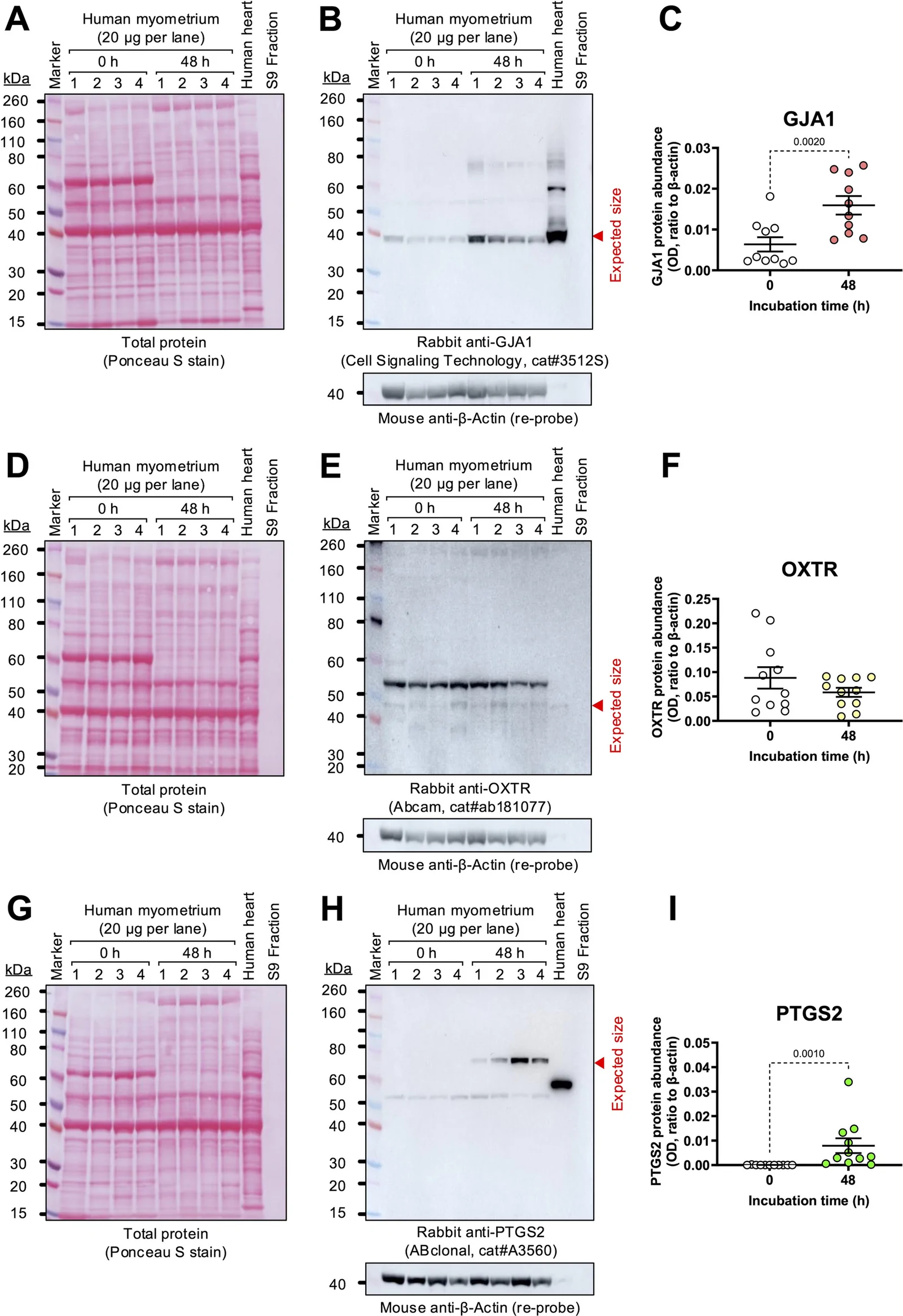
Protein level changes of GJA1, OXTR, and PTGS2 when term pregnant non-laboring human myometrium was cultured for 48 h ex vivo*.* Protein was extracted from term non-laboring myometrium samples immediately after biopsy (0 h) (*n* = 11) or following 48 h incubation ex vivo (*n* = 11), separated by SDS-PAGE (20–50 µg/lane), and then transferred to a nitrocellulose membrane. S9 Fraction from human liver extract (0.5 µg/lane) and human heart extract (20 µg/lane) were included as negative and positive controls. Total protein transfer was visualized by Ponceau S staining and then imaged (panels **A**, **D**, **G**). Membranes were then probed for GJA1 (Cell Signaling Technology, cat#3512S, 1:1,000), OXTR (Abcam, cat#ab181077, 1:1,000), PTGS2 (ABclonal, cat#A3560, 1:1,000), and anti-rabbit-HRP secondary antibody (1:2,000). Representative images show immunoreactive bands detected using Immobilon Forte chemiluminescence reagent (panels **B**, **E**, **H**). Blots were then stripped and re-probed using mouse anti-β-actin (1:4,000) and anti-mouse-HRP (1:2,000). The molecular weight marker was Novex™ Sharp Pre-stained Protein Standard. The optical density of the immunoreactive band corresponding to the expected molecular weight of GJA1 (39 kDa), OXTR (43 kDa), PTGS2 (72 kDa), and β-actin (42 kDa) was measured, then GJA1, OXTR, and PTGS2 protein abundance was expressed relative to β-actin (panels **C**, **F**, **I**). Data were checked using the Shapiro–Wilk normality test and analyzed by paired *t*-test. Data are mean ± SEM. A significant *p*-value is indicated

Following 48 h culture, significant increases in protein levels were observed for the CAPS, GJA1 (*p* = 0.0020) and PTGS2 (*p* = 0.0010), whereas OXTR protein levels trended toward decreasing but did not reach statistical significance (*p* = 0.1292) (Fig. 5).

**Fig. 6:**
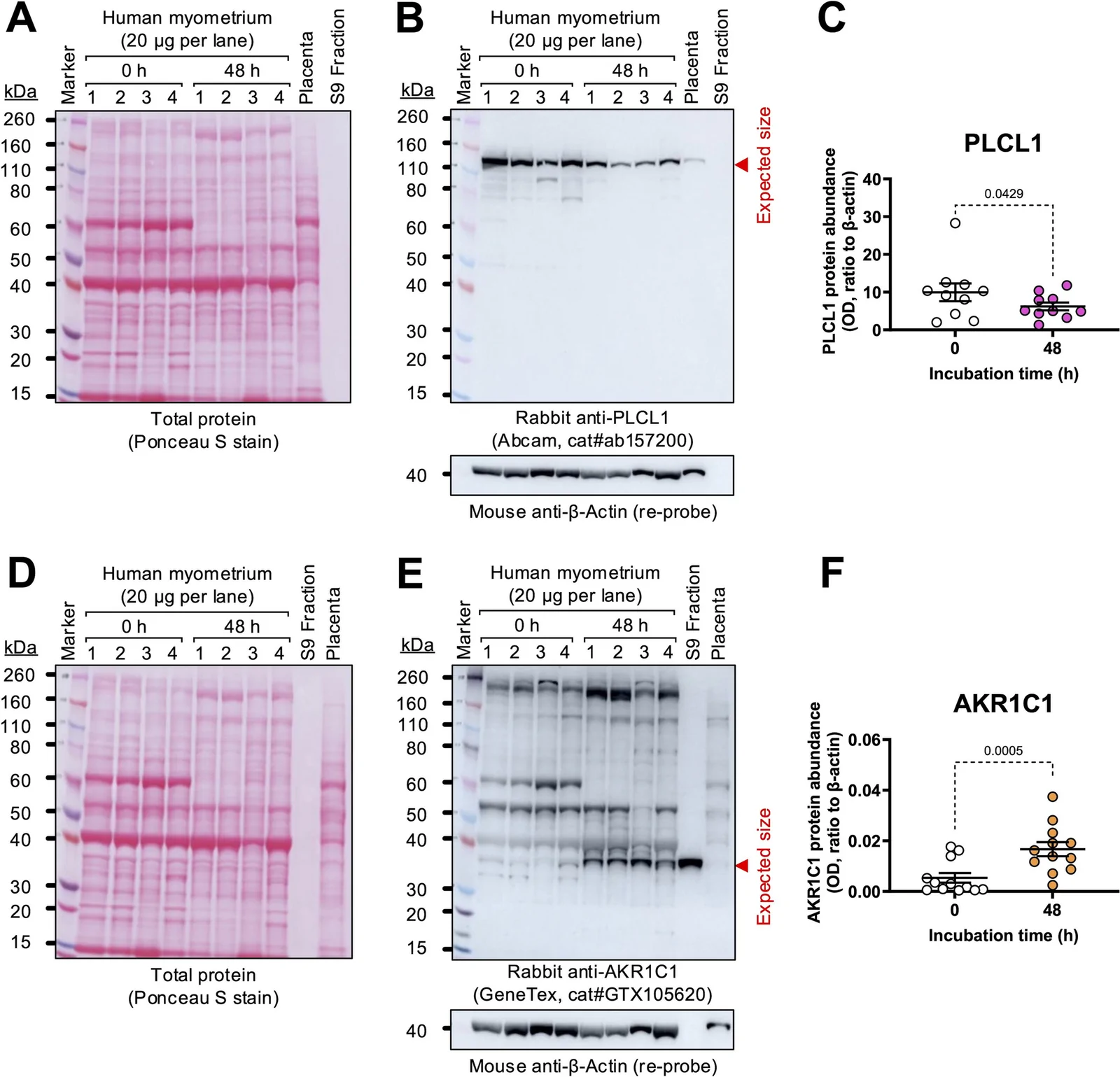
Protein level changes of PLCL1 and AKR1C1 when term pregnant, non-laboring human myometrium was cultured for 48 h ex vivo*.* Protein was extracted from term non-laboring myometrium samples immediately after biopsy (0 h) (*n* = 10–12) or following 48 h incubation ex vivo (*n* = 10–12), separated by SDS-PAGE (20–50 µg/lane), and then transferred to a nitrocellulose membrane. S9 Fraction from human liver extract (0.5 µg/lane) and human placenta extract (20 µg/lane) were included as positive and negative controls. Total protein transfer was visualized by Ponceau S staining and then imaged (panels **A** and **D**). Membranes were then probed for PLCL1 (Abcam, cat#ab157200, 1:1,000), AKR1C1 (GeneTex, cat#GTX105620, 1:1,000), and anti-rabbit-HRP secondary antibody (1:2,000). Representative images show immunoreactive bands detected using Immobilon Forte chemiluminescence reagent (panels **B** and **E**). Blots were then stripped and re-probed using mouse anti-β-actin (1:4,000) and anti-mouse-HRP (1:2,000). The molecular weight marker was Novex™ Sharp Pre-stained Protein Standard. The optical density of the dominant immunoreactive band corresponding to the expected molecular weight of PLCL1 (123 kDa) and AKR1C1 (37 kDa) was measured, and protein abundance was expressed relative to β-actin (panels **C** and **F**). Data were checked using the Shapiro–Wilk normality test and analyzed by paired *t*-test. Data are mean ± SEM. A significant *p*-value is indicated

Of the assessed enzymes, PLCL1 protein levels significantly decreased (*p* = 0.0429) while AKR1C1 protein levels significantly increased (*p* = 0.0005) following 48 h culture (Fig. 6). As previously noted, however, [20, 34], the utilized GeneTex anti-AKR1C1 antibody (as well as other available AKR1C1 antibodies) lacks specificity for AKR1C1 due to extensive amino acid sequence homology with AKR1C2 (97%) and AKR1C3 (88%). Nonetheless, the immunoreactive band analyzed was the appropriate molecular weight for AKR1C1 (37 kDa).

**Fig. 7:**
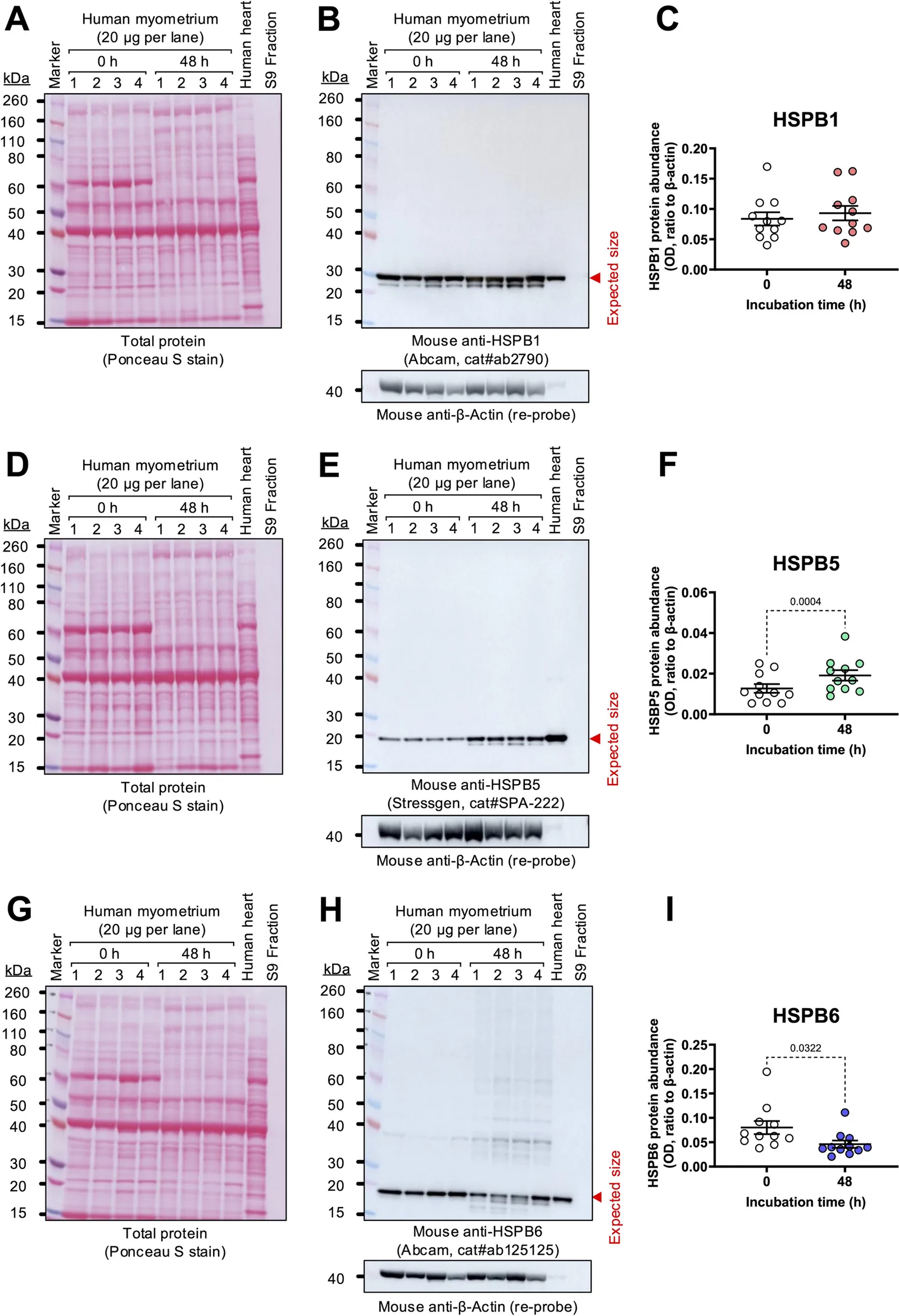
Protein level changes of HSPs when term pregnant, non-laboring human myometrium was cultured for 48 h ex vivo*.* Protein was extracted from term non-laboring myometrium samples immediately after biopsy (0 h) (*n* = 11) or following 48 h incubation ex vivo (*n* = 11), separated by SDS-PAGE (20–50 µg/lane), and then transferred to a nitrocellulose membrane. S9 Fraction from human liver extract (0.5 µg/lane) and human heart extract (20 µg/lane) were included as positive and negative controls. Total protein transfer was visualized by Ponceau S staining and then imaged (panels **A**, **D**, **G**). Membranes were then probed for HSPB1 (Abcam, cat#ab2790, 1:2,500), HSPB5 (Stressgen, cat#SPA-222, 1:1,000), HSPB6 (Abcam, cat#ab125125, 1:1,000), and anti-mouse-HRP secondary antibody (1:2,000). Representative images show immunoreactive bands detected using Immobilon Forte chemiluminescence reagent (panels **B**, **E**, **H**). Blots were then stripped and re-probed using mouse anti-β-actin (1:4,000) and anti-mouse-HRP (1:2,000). The molecular weight marker was Novex™ Sharp Pre-stained Protein Standard. The optical density of the dominant immunoreactive band corresponding to the expected molecular weights of HSPB1 (27 kDa), HSPB5 (20 kDa), and HSPB6 (17 kDa) was measured, and protein abundance was expressed relative to β-actin (panels **C**, **F**, **I**). Data were checked using the Shapiro–Wilk normality test and analyzed by paired *t*-test. Data are mean ± SEM. A significant *p*-value is indicated

Analysis of the HSPs revealed that following 48 h culture, HSPB1 protein levels remained unchanged (*p* = 0.3453), whereas HSPB5 protein levels significantly increased (*p* = 0.0004), while HSPB6 protein levels significantly decreased (*p* = 0.0322) (Fig. 7).

## Discussion

We have previously shown that key labor-associated genes, including *ESR1*, *PTGS2*, *OXTR*, and *PGR*, undergo significant changes in expression that are consistent with labor onset in vivo when pregnant human myometrial explants are incubated for 48 h ex vivo [18, 19]. Using this model, we further documented a significant increase in *AKR1C1* mRNA abundance over the 48 h culture period, also consistent with labor onset in vivo [33–35]. In this study, we examined the expression of numerous additional key genes that undergo significant expression changes during labor onset in vivo, including CAPs, upstream regulators, inflammatory mediators, enzymes, and small HSPs, to validate that culturing pregnant human myometrium as explants for 48 h constitutes an ‘ex vivo labor model’.

Our study showed that CAP-encoding gene expression was significantly upregulated, whereas RAP-encoding gene expression was significantly downregulated following 48 h of ex vivo incubation. The observed changes are consistent with gene expression changes associated with term labor onset in vivo. These findings suggest that when the term not-in-labor myometrium is cultured ex vivo, it transitions to a labor-like state, supporting the application of this model as an ex vivo model of the onset of human labor.

Tissue incubation studies are routinely performed for 48 h or more [37, 44, 45]. Among these, myometrial strips obtained from non-laboring pregnant women can develop spontaneous contractile activity within 1–2 h when suspended in organ baths under tension [46–49]. However, spontaneous contractions are not specific to pregnancy or labor, as spontaneous activity has also been extensively documented in ex vivo myometrial strips obtained from non-pregnant women [50–53]. Accordingly, the development of spontaneous contractions following tissue isolation likely reflects, at least in part, the intrinsic contractile properties of human myometrium and should not in isolation be interpreted as evidence of a labor-associated phenotypic transition. In the present study, evidence for acquisition of a labor-like phenotype is instead based on the coordinated molecular remodeling observed across multiple pathways implicated in human labor.

ESR1 (ERα) plays a central upstream regulatory role in human labor by mediating estrogen-induced activation of the myometrium, thus promoting the transition from quiescence to a contractile phenotype. It does this by increasing gap junction formation for synchronization, enhancing prostaglandin production and sensitivity, and promoting inflammation via cytokines such as TNFα, IL1β, IL6, and IL8. We found that *ESR1* expression increased significantly after 48 h of ex vivo incubation. This is consistent with previous research showing that *ESR1* increased significantly at labor onset in the pregnant human myometrium at term [32, 37]. Since *ESR1* regulates many CAPs, we assessed the expression of *GJA1*, a major gap junction protein essential for synchronized contractions, and *PTGS2*, a key enzyme in prostaglandin synthesis that increases local production of PGE_2_ and PGF_2α_, thereby reinforcing contractile activity, and *OXTR*, the G protein-coupled receptor responsible for oxytocin-mediated augmentation of myometrial contractility. Within our ex vivo labor model, both *GJA1* and *PTGS2* expression increased significantly, consistent with labor onset in vivo [23, 27, 30, 36], whereas *OXTR* expression decreased significantly, also consistent with labor onset in vivo [21, 24, 29, 30, 36]. Our observed significant increase in *PTGS1* expression is inconsistent with prior reports that its expression remains stable throughout human pregnancy and labor [27, 54]; however, PTGS1 has been shown not to play a major role in the labor cascade in humans [27, 54].

*HPGD* encodes 15-hydroxyprostaglandin dehydrogenase (15-PGDH), the primary enzyme responsible for the initial inactivation of prostaglandins, particularly PGE_2_ and PGF_2α_, by converting them to inactive 15-keto metabolites, thus promoting myometrial quiescence during pregnancy. Although *HPGD* mRNA abundance increased in the ex vivo labor model, against expectations, it remains to be established whether *HPGD* undergoes any significant up- or downregulation in association with labor onset in vivo. Therefore, we cannot at this stage confirm whether the observed upregulation of *HPGD* expression in our ex vivo labor model is consistent with labor onset in vivo.

Previously, we showed that *PR-A* expression increased significantly after 48 h of incubation of human myometrial explants, whereas *PR-B* expression remained unchanged, resulting in a significant increase in the *PR-A*/*PR-B* ratio [19]. This is consistent with changes in *PR* isoform expression that occur during labor onset in vivo [26, 28, 32]. As such, we also sought to examine the expression of *AKR1C1* and *SRD5A1*, as key enzymes involved in progesterone metabolism in the myometrium. *AKR1C1* contributes to functional progesterone withdrawal by converting active progesterone to its inactive metabolite, 20α-hydroxyprogesterone (20α-OHP), thereby reducing progesterone availability for the pro-quiescence activation of progesterone receptor-B (PR-B) and unliganding pro-labor PR-A, which, together, allow upregulated expression of CAPs, such as *GJA1*, *OXTR*, and *PTGS2* [35, 55]. *AKR1C1* expression is significantly upregulated in our ex vivo labor model, consistent with previous studies showing that myometrial *AKR1C1* mRNA abundance and 20α-HSD protein levels increase with the onset of human labor at term [25, 31, 33–35]. *SRD5A1* encodes Steroid 5 Alpha-Reductase 1, which converts progesterone to 5α-dihydroprogesterone (5α-DHP), another inactive metabolite. Myometrial *SRD5A1* expression increased significantly in the ex vivo labor model, consistent with SRD5A1 promoting progesterone withdrawal and labor onset; however, this is inconsistent with a previous report that SRD5A1 remains unchanged with labor onset in humans [25]. Additional comparisons of *SRD5A1* expression in non-laboring versus laboring pregnant human myometrium are necessary to clarify whether SRD5A1 expression changes in association with labor onset in vivo.

MED17 is a subunit of the Mediator complex, a large multi-protein coactivator that bridges transcription factors to RNA polymerase II and regulates gene transcription in eukaryotes. The Mediator complex is crucial for integrating signals from transcription factors such as NF-κB (inflammation), ESR1 (estrogen activation), and *PR* isoforms, which orchestrate myometrial activation during labor. However, as there is currently no evidence of significant upregulation or downregulation of myometrial *MED17* expression in association with human labor onset in vivo, we could not confirm whether the upregulated *MED17* expression observed in our ex vivo labor model is consistent with labor onset in vivo.

NFκB3 (p65/RELA) is a subunit of the NFκB complex, activation of which is a central event in the inflammatory switch that transforms the quiescent myometrium into a contractile laboring state [56]. It regulates the expression of immune/inflammatory genes, including cytokines such as *TNFα*, *IL1β*, *IL6*, and *IL8*, as well as CAP-encoding genes, such as *GJA1*, *OXTR*, and *PTGS2* [56]. The “canonical” NFκB complex is a heterodimer of NFκB3 (p65/RELA) and NFκB1 (p50) [57]. NFκB3 is the main driver of transcriptional activation, while NFκB1 is essential for DNA binding and dimer stability [57]. Previous research showed a significant decrease in the overall protein levels of both NFκB1 and NFκB3 in the lower but not the upper uterine segment during labor [57]. Moreover, NFκB3 was primarily cytoplasmic before labor and nuclear during labor in the upper segment of the myometrium [58]. Currently, there is no strong evidence of significant upregulation or downregulation of *NFκB1* and *NFκB3* expression specifically at the transition to labor in human myometrium. Therefore, we could not confirm whether the upregulated myometrial expression of *NFκB1* and *NFκB3* observed in our ex vivo labor model is consistent with labor onset in vivo.

TNFα is a key pro-inflammatory cytokine that plays a significant role in the inflammatory activation of the human myometrium during labor. It activates the NF-κB pathway, which leads to upregulation of CAPs, chemokines that recruit immune cells, and *PR-A*, thereby inducing functional progesterone withdrawal [59]. Moreover, TNFα synergizes with other cytokines, such as IL1β, and with mechanical stretch to sustain the inflammatory milieu [59]. We observed a significant increase in myometrial *TNFα* expression in our ex vivo labor model, consistent with previous research showing that *TNFα* levels rise markedly at labor onset in vivo [34, 45–48].

The pro-inflammatory cytokines IL1β, IL6, and IL8 play central roles in the inflammatory activation of the human myometrium during labor. IL1β is the most potent initiator of labor-associated inflammation. It stimulates prostaglandin production via upregulating *PTGS2* expression [60], enhancing calcium signaling for contractions [61], promoting *PR* changes [62], inducing the production of other cytokines/chemokines [63, 64], and activating NFκB pathways [65, 66]. IL6 is a key trigger and amplifier in the labor cascade, as it stimulates prostaglandin and oxytocin production [64, 67], facilitates cervical ripening [68], indirectly induces uterine contractions [64, 67], and promotes immune-endocrine crosstalk [69, 70]. IL8 drives neutrophil and monocyte chemotaxis and recruitment to the myometrium, amplifying local inflammation [71, 72], and promotes leukocyte infiltration, which releases additional pro-labor mediators (cytokines, reactive oxygen species, prostaglandins) [73]. IL8, therefore, indirectly enhances myometrial activation via immune cell influx and is a downstream target of the NFκB/activator protein-1 (AP-1) pathways. Along with TNFα, these cytokines form part of a coordinated “core inflammatory response”. Accordingly, prior studies have shown that these pro-inflammatory cytokines increase significantly at the onset of labor in vivo [30, 36, 74–76]. Reassuringly, we observed that expression of *IL1β*, *IL6*, and *IL8* all significantly increased in our ex vivo labor model, consistent with labor onset in vivo.

*PLCL1* and *PLCL2* are key negative regulators of intracellular calcium signaling by sequestering inositol 1,4,5-trisphosphate (IP_3_) or modulating phosphatidylinositol 4,5-bisphosphate (PIP_2_) metabolism, thereby attenuating calcium release from intracellular stores and suppressing smooth muscle contraction [77]. Previous research has shown that *PLCL1* predominates in the human myometrium, whereas Plcl2 is more prominent in the pregnant mouse uterus; nevertheless, both are present in the human myometrium [78]. Consistent with previous research, we detected both *PLCL1* and *PLCL2* mRNA in term, non-laboring pregnant human myometrium, with *PLCL1* exhibiting higher baseline abundance than *PLCL2*. Furthermore, we observed that *PLCL1* mRNA abundance and PLCL1 protein levels decreased significantly within our ex vivo labor model, which is consistent with labor onset in vivo [30, 36, 74]. Currently, there is no strong evidence of significant upregulation or downregulation of *PLCL2* mRNA abundance or PLCL2 protein levels specifically at the transition to labor in human myometrium. Therefore, we could not confirm whether the upregulated expression of *PLCL2* observed in our ex vivo labor model is consistent with labor onset in vivo.

TRPV4 is a mechanosensitive, calcium-permeable ion channel expressed in human myometrial smooth muscle cells. Previous research has shown that *TRPV4* mRNA abundance significantly increases with advancing gestation (mid- to late pregnancy) [79]; however, there is no consistent evidence establishing either a dramatic further increase or a decrease at labor onset (laboring vs. non-laboring term myometrium). Therefore, we could not confirm whether the significant increase in *TRPV4* expression observed in our ex vivo labor model is consistent with labor onset in vivo. However, as TRPV4 activation leads to calcium influx and activation of the canonical calcium-dependent contraction signaling pathway, upregulated TRPV4 expression, as observed in our model, is generally considered pro-labor.

The myometrium undergoes significant remodeling during labor, for instance, cytoskeletal reorganization, increased excitability, inflammation, and mechanical stress from uterine distension/stretch. Small HSPBs play crucial roles in protecting cells from stress by stabilizing actin filaments, regulating actin-myosin interactions, and responding to oxidative/inflammatory cues, thus supporting the contractile apparatus without being classic CAPs [80]. Although *HSPB1* mRNA abundance significantly decreased in our ex vivo labor model, HSPB1 protein levels remained unchanged, which is consistent with previous research showing that HSPB1 protein levels remained unchanged during labor onset [41]. *HSPB5* mRNA abundance and HSPB5 protein levels both significantly increased in our ex vivo labor model, which is in contrast to a previous report that HSPB5 protein levels decreased with labor onset [41]. Finally, *HSPB6* mRNA abundance and HSPB6 protein levels decreased significantly in our model; this is consistent with RNA-Seq data showing a significant decrease in *HSPB6* mRNA abundance with labor [34], but inconsistent with protein data, which showed HSPB6 protein levels remained unchanged [49]. Currently, there is no strong evidence of significant upregulation or downregulation of *HSPB1* and *HSPB5* mRNA abundance at the transition to labor in human myometrium. Therefore, we could not confirm whether the changes in expression of these small HSPBs observed in our ex vivo labor model are consistent with labor onset in vivo.

PDEs are enzymes that hydrolyze cyclic nucleotides, primarily cAMP and cGMP, which are key second messengers that promote myometrial relaxation during pregnancy by activating protein kinase A (PKA) and protein kinase G (PKG), respectively, thereby reducing intracellular calcium and inhibiting contractions. PDE4 is the largest PDE family known to date, encoded by four distinct genes, PDE4A, PDE4B, PDE4C, and PDE4D. All four *PDE4* variants have been found in pregnant myometrium, with *PDE4D* most prominently expressed, *PDE4A* and *PDE4B* markedly abundant, and *PDE4C* expressed at low levels [81]. Interestingly, *PDE4B2* expression was more abundant in the myometria of pregnant women compared to non-pregnant women, whereas no difference was detected for *PDE4A*, *PDE4C*, and *PDE4D* between the two tissues [81]. Currently, there is no strong evidence of significant upregulation or downregulation of *PDE4* mRNA abundance at the transition to labor in human myometrium. Therefore, we could not confirm whether the significantly upregulated expression of *PDE4A, PDE4B, PDE4C,* and *PDE4D* observed in our ex vivo labor model is consistent with labor onset in vivo. PDE5 is a major cGMP-hydrolyzing enzyme and is responsible for an estimated 75% of the total cGMP-hydrolyzing activity [82]. Belmonte et al. [83] detected PDE5 protein in human myometrial cells throughout pregnancy, with no major change reported with advancing gestation or at labor onset; however, the expression of *PDE5* mRNA has yet to be explored in the human myometrium. Thus, we could not confirm whether the increased *PDE5A* expression observed in our ex vivo labor model is consistent with labor onset in vivo. PDE7, a specific cAMP-hydrolyzing enzyme encoded by two genes (*PDE7A* and *PDE7B*), was found in vascular smooth muscle where it regulates cAMP signaling involved in inflammation, cell proliferation, and contractility [84, 85]. Within the current literature, PDE7 has minimal to no documented role in human myometrium or labor onset. Here we report the detection of both *PDE7A and PDE7B* mRNA in non-laboring pregnant human myometrium, and that their expression significantly increased in our ex vivo labor model. Further research is needed to determine whether PDE7 isoforms play a role in the myometrium during labor onset in vivo. Finally, PDE8 is another specific cAMP-hydrolyzing enzyme with two isoforms, PDE8A and PDE8B. Based on current literature, PDE8 has minimal to no established role in human labor/myometrium. Nevertheless, an RNA-Seq study by Chan et al. [36] reported a significant decrease in *PDE8B* expression with labor onset in vivo. We detected both *PDE8A* and *PDE8B* mRNA in non-laboring pregnant human myometrium, with *PDE8A* expression significantly increasing in our ex vivo labor model, whereas *PDE8B* expression remained unchanged. Further research is needed to examine the expression of PDE8 isoforms in the human myometrium and to determine whether they promote labor onset.

In summary, combining the current dataset with our previously published *PR-A* and *PR-B* findings from the same explant model, 17 targets could be compared with established in vivo labor-associated changes. Fourteen exhibited consistent changes in expression in our ex vivo labor model. These 14 genes were *ESR1, GJA1*, *OXTR*, *PTGS2*, *PR-A*,* PR-B*, *AKR1C1*, *TNFα*, *IL1β*, *IL6*, *IL8*, *PLCL1*, *HSPB1*, and HSPB6. Only *PTGS1*, *SRD5A1*, and *PDE8B* exhibited expression changes that may be inconsistent with labor onset in vivo; however, these genes have been shown to play more secondary or compensatory roles in human labor. The other 15 genes examined in our ex vivo labor model have no established expression in the human myometrium or role in labor onset; therefore, we could not confirm whether the expression changes we observed in our model are consistent with labor onset in vivo. The results are summarized in Table 3.

Whole-transcriptome analysis conducted by Georgiou et al*.* [15] demonstrated that although explants cultured for 30 h more closely matched the gene expression profile of freshly biopsied myometrium than cultured primary or immortalized cells, the explants still exhibited significant expression changes in key genes relevant to myometrial function and parturition. These included increased expression of *PTGS2* (7.02 ± 3.34 fold-change), *IL6* (20.12 ± 4.27 fold-change), *IL8* (200.53 ± 43.89 fold-change), *IL1β* (2.53 ± 0.09 fold-change), and *GJA1* (1.06 ± 0.18 fold-change), and decreased expression of *OXTR* (− 11.93 ± 0.05 fold-change) [15]. We observed consistent gene expression changes in our 48 h cultured myometrial explants and documented many additional changes.

The mechanisms responsible for this culture-induced molecular remodeling remain to be established and are likely multifactorial. Removal of the myometrium from its native environment abruptly alters several physiological inputs that normally contribute to the maintenance of pregnancy. For example, explants are no longer exposed to the normal circulating endocrine milieu, including sustained progesterone exposure, and are separated from systemic and neighboring tissue-derived signals. Although the explant retains its local multicellular architecture and therefore preserves some cell–cell and paracrine interactions, the absence of vascular perfusion necessarily alters oxygen, nutrient, metabolite, and waste-product gradients. Adaptation to defined serum-free culture medium may further modify cellular metabolism and signaling. In addition, biopsy collection and dissection into small explants impose tissue injury and remove the mechanical forces experienced by the myometrium within the gravid uterus, either of which could initiate cellular stress and inflammatory responses. Although the precise underlying mechanism(s) are yet to be determined, the engagement of pathways that overlap with the physiological processes accompanying labor, including inflammatory signaling, functional progesterone withdrawal, prostaglandin synthesis, and cellular stress responses, suggests this model may nonetheless have experimental utility.

Taken together, these findings support the interpretation that explants of non-laboring myometrium undergo coordinated remodeling toward a labor-like molecular phenotype during 48 h of ex vivo culture. We therefore propose this system as an ex vivo model of the molecular transition toward human labor, while recognizing that direct comparison with laboring tissue and functional validation are still required.

This phenomenon has two important implications. First, data generated from prolonged culture of supposedly ‘non-laboring’ myometrial explants may no longer faithfully represent the freshly biopsied non-laboring state, which could confound interpretation of experiments intended to investigate quiescent myometrial biology. Second, the reproducible acquisition of labor-associated molecular characteristics creates an experimentally accessible system for interrogating mechanisms that drive the transition of term human myometrium toward a labor-like state.

Collectively, these findings move the field beyond viewing culture-induced divergence in myometrial explants simply as experimental drift. Instead, prolonged culture of term non-laboring human myometrium appears to represent a biologically informative transition state. This both exposes an under-recognized limitation of conventional explant studies and provides a new experimental opportunity to interrogate the temporal and mechanistic events associated with myometrial activation that precede established labor and are difficult to study directly in pregnant women.

The potential utility of this model extends beyond providing an alternative source of tissue exhibiting labor-associated characteristics. Comparisons of freshly collected non-laboring and laboring myometrium provide cross-sectional snapshots of two clinical states, whereas the present model begins with non-laboring tissue and enables the experimental examination of the transition toward a labor-like molecular phenotype within tissue from the same participant. This creates opportunities to define the temporal sequence of myometrial activation, identify early-response pathways, test candidate triggers and inhibitors, and determine whether the transition can be prevented or reversed. The explant system also retains multicellular composition, extracellular matrix, and three-dimensional tissue architecture that are absent from conventional monolayer cell cultures.

A major strength of this study is the use of primary term, non-laboring pregnant human myometrium, which provides greater physiological relevance to human parturition than isolated primary cells, immortalized cell lines, or animal models. The paired experimental design, in which freshly biopsied tissue from each participant served as the 0 h comparator for explants cultured for 48 h, minimizes the influence of inter-individual variability and allows culture-induced changes to be assessed within the same biological sample. The relatively large cohort used for the gene expression analyses (*n* = 30) further strengthened the robustness of these comparisons. Importantly, the study examined a broad panel of genes spanning multiple biological processes implicated in labor, including myometrial contractility, steroid and prostaglandin signaling, inflammation, calcium regulation, cellular stress, and cyclic nucleotide signaling, rather than relying on changes in a small number of canonical contraction-associated proteins. Many of the observed changes were concordant with those previously reported during the transition to labor in vivo, and selected findings were additionally assessed at the protein level. Together, these features provide convergent evidence that term non-laboring myometrial explants undergo coordinated molecular remodeling during ex vivo culture toward a labor-like phenotype, with numerous potential experimental applications, as previously defined.

Several limitations should nevertheless be considered when interpreting these findings. First, the comparison was made only between fresh (0 h) and 48 h-cultured explants. Future directions therefore include assessing the model over a more comprehensive timeline, including shorter incubation time points (e.g. 1, 2, 6, & 24 h), to determine how rapidly phenotypic transformation occurs (and potentially highlight the early responder genes that may be driving the transition), as well as longer timepoints (72, 96, & 120 h) to determine whether changes in gene expression and/or protein levels are transient or sustained. Second, the present study establishes a labor-like molecular phenotype but does not demonstrate a corresponding functional transition. Future studies would therefore ideally determine whether the documented changes in labor-associated gene and protein expression are accompanied by alterations in spontaneous contractile activity and responsiveness to uterotonics; however, such contractile studies are typically conducted with tissue strips or rings, and would require adaptation of existing organ bath equipment to be compatible with assessing the contractility of small, roughly cuboidal myometrial explants (~ 2 × 2 × 2 mm). Third, the analysis includes a targeted panel of 30 specific genes. Additional future directions, therefore, include omics analyses comparing explants across the ex vivo incubation timeline against freshly biopsied non-laboring and laboring pregnant human myometrium. Such analyses would provide valuable insights into how broadly our ex vivo labor model recapitulates the onset of labor in vivo. Fourth, inflammatory mediators were assessed at the mRNA level only. Transcriptome-wide analyses and measurement of cytokine protein in tissue or culture supernatants would provide broader validation of the model. Fifth, the removal of tissue from its native environment and subsequent ex vivo culture necessarily alter endocrine, paracrine, metabolic, mechanical, and oxygen-related inputs, and tissue injury or culture-associated stress may contribute to some of the observed transcriptional changes. Tissue viability, apoptosis, and cellular stress were not directly assessed and should be factored into future studies. Finally, the study population was heterogeneous with respect to clinical and maternal characteristics, including gestational age, BMI, obstetric indication for cesarean delivery, and potentially medication exposure. These factors may influence baseline myometrial phenotype or the magnitude of culture-induced responses. Although the paired design minimizes the influence of inter-individual variability on the primary analysis, the study was not designed to determine whether maternal or obstetric characteristics modify the magnitude or trajectory of the ex vivo transition.

In conclusion, term, non-laboring pregnant human myometrial explants undergo substantial molecular remodeling during 48 h of ex vivo culture, with coordinated changes in contractile, inflammatory, steroid-regulatory, prostaglandin, calcium-signaling, and cyclic nucleotide pathways. Many of these changes are consistent with those reported during the transition to labor in vivo, supporting the concept that cultured non-laboring myometrium acquires a labor-like molecular phenotype. These findings identify an important limitation of prolonged myometrial explant culture for studies intended to model the non-laboring state, while also establishing a potentially valuable ex vivo system for investigating mechanisms underlying human myometrial activation and labor onset.

## Data Availability

Data available on request due to privacy/ethical restrictions.

## Glossary of Terms

1D: One-dimensional
2D: Two-dimensional
3D: Three-dimensional
15-PGDH: 15-Hydroxyprostaglandin dehydrogenase
20α-HSD: 20α-Hydroxysteroid dehydrogenase
20α-OHP: 20α-Hydroxyprogesterone
5α-DHP: 5α-Dihydroprogesterone
AKR1C1: Aldo-keto reductase family 1 member C1 gene
ANOVA: Analysis of variance
AP-1: Activator protein-1
BCA: Bicinchoninic acid
cAMP: Cyclic adenosine monophosphate
cGMP: Cyclic guanosine monophosphate
CAP: Contraction-associated protein
CS: Cesarean section
Cx43: Connexin 43
EDTA: Ethylenediaminetetraacetic acid
DMEM: Dulbecco’s modified Eagle medium
E2: Estradiol
ESR1: Estrogen receptor 1
GJA1: Gap junction alpha-1 protein
HPGD: 15-Hydroxyprostaglandin dehydrogenase
HSPB1: Heat shock protein family B (small) member 1
HSPB5: Heat shock protein family B (small) member 5
HSPB6: Heat shock protein family B (small) member 6
HRP: Horseradish peroxidase
hTERT: Human telomerase reverse transcriptase
IL1β: Interleukin 1 beta
IL6: Interleukin 6
IL8: Interleukin 8
IP3: Inositol 1,4,5-trisphosphate
MED17: Mediator of RNA polymerase II transcription subunit 17
mRNA: Messenger ribonucleic acid
NFκB1: Nuclear factor kappa-B 1
NFκB3: Nuclear factor kappa-B 3
OXTR: Oxytocin receptor gene
P4: Progesterone
PBS: Phosphate-buffered solution
PDE4A: Phosphodiesterase 4A
PDE4B: Phosphodiesterase 4B
PDE4C: Phosphodiesterase 4C
PDE4D: Phosphodiesterase 4D
PDE5A: Phosphodiesterase 5A
PDE7A: Phosphodiesterase 7A
PDE7B: Phosphodiesterase 7B
PDE8A: Phosphodiesterase 8A
PDE8B: Phosphodiesterase 8B
PG: Prostaglandin
PGF_2α_: Prostaglandin F2α
PIP2: Phosphatidylinositol 4,5-bisphosphate
PKA: Protein kinase A
PKG: Protein kinase G
PLCL1: Phospholipase C like 1
PLCL2: Phospholipase C like 2
PR: Progesterone receptor
RELA: V-rel avian reticuloendotheliosis viral oncogene homolog A
RAP: Relaxation-associated protein
RT-PCR: Real-time quantitative polymerase chain reaction
PTGS1: Prostaglandin-endoperoxide synthase 1
PTGS2: Prostaglandin-endoperoxide synthase 2
RNA: Ribonucleic acid
SMC: Smooth muscle cell
SDS: Sodium dodecyl sulfate
SDS-PAGE: Sodium dodecyl sulfate–polyacrylamide gel electrophoresis
SRD5A1: Steroid 5-alpha-reductase 1
TBS: Tris buffered saline
TBS-T: Tris buffered saline tween-20
TNFα: Tumor necrosis factor alpha
TRPV4: Transient receptor potential cation channel subfamily V member 4
